# The postoperative bleeding rate and its risk factors in patients on antithrombotic therapy who undergo gastric endoscopic submucosal dissection

**DOI:** 10.1186/1471-230X-13-136

**Published:** 2013-09-06

**Authors:** Toshihisa Takeuchi, Kazuhiro Ota, Satoshi Harada, Shoko Edogawa, Yuichi Kojima, Satoshi Tokioka, Eiji Umegaki, Kazuhide Higuchi

**Affiliations:** 1 2nd Department of Internal Medicine, Osaka Medical College, 2-7 Daigaku-machi, Takatsuki 569-8686, Osaka, Japan

**Keywords:** Antithrombotic agents, Endoscopic submucosal dissection, Gastroprotective agent, Peptic ulcer, Proton-pump inhibitor

## Abstract

**Background:**

There is a lack of consensus regarding the risk of postoperative hemorrhage in patients on antithrombotic therapy who undergo endoscopic submucosal dissection (ESD).

We examined postoperative bleeding rates and risk factors for postoperative hemorrhage from post-ESD gastric ulcers in patients on antithrombotic therapy.

**Methods:**

The subjects of this study were 833 patients who underwent ESD of gastric tumors. Of these, 743 were not on antithrombotic therapy and 90 were on some form of antithrombotic therapy (46 on low-dose aspirin (LDA) only, 23 on LDA + thienopyridine, and 21 on LDA + warfarin). All patients commenced proton pump inhibitor (PPI) therapy immediately postoperatively. Antiplatelet agents were discontinued for 7 days preoperatively and postoperative Day 1, and anticoagulants for 5 days preoperatively and postoperative Day 1.

**Results:**

The postoperative bleeding rate in the antithrombotic group was 23.3%, significantly higher than the 2.0% observed in the non-antithrombotic group. Significant differences were seen in patients in the antithrombotic group with and without postoperative bleeding according to ESD duration (p = 0.041), PPI + mucosal protective agent combination therapy (p = 0.039), and LDA + warfarin combination therapy (p < 0.001). Multivariate analysis of these factors yielded odds ratios of 1.04 for ESD duration, 14.83 for LDA + warfarin combination therapy, and 0.27 for PPI + mucosal protective agent combination therapy.

**Conclusions:**

The risk of postoperative hemorrhage following gastric ESD was higher in patients with antithrombotic therapy than in those without that therapy. Among these patients, LDA + warfarin combination therapy and longer ESD duration were significant risk factors for postoperative bleeding. On the contrary, a mucosal protective agent to PPI therapy, lowering the odds ratio for postoperative bleeding, which suggests that the addition of a mucosal protective agent might be effective in preventing post-ESD hemorrhage in patients on antithrombotic therapy.

## Background

Endoscopic submucosal dissection (ESD) is increasingly used worldwide in the treatment of gastric tumors. However, postoperative complications of ESD, including perforations of the upper gastrointestinal tract and post-ESD ulcer bleeding (postoperative hemorrhage), are increasingly becoming a problem. Whereas perforations are mainly a problem with technique, postoperative hemorrhage is a serious complication that occurs in a certain proportion of patients irrespective of technical considerations. Studies concerning post-ESD ulcer healing and postoperative hemorrhage have reported that proton pump inhibitor (PPI) therapy gives good healing rates for post-ESD ulcers, and that it is also effective in preventing postoperative hemorrhage [1,2], so PPIs are widely administered post-ESD.

With the advance of the ageing society, we are increasingly likely to perform ESD in patients with concurrent medical conditions, in particular heart conditions and cerebrovascular disease. Many of these patients are on long-term antithrombotic therapy (antiplatelet agents or anticoagulants). Patients on antiplatelet agents such as low-dose aspirin (LDA) have a greater risk and frequency of upper gastrointestinal hemorrhage, and Luis et al. [3] reported adjusted relative risks for upper gastrointestinal hemorrhage of 1.79 for LDA monotherapy, 3.71 for LDA + thienopyridine, and 3.62 for LDA + anticoagulant combination therapy. Furthermore, inhibitors of acid secretion such as PPIs have been reported to be effective in reducing the incidence and prevalence of upper gastrointestinal hemorrhage [4-6].

For less invasive endoscopic procedures such as biopsies, the risk of bleeding increases very little in patients taking antiplatelet agents [7-9], and even in patients on anticoagulant therapy the risk of postoperative hemorrhage is unchanged as long as the prothrombin time-international normalized ratio (PT-INR) is under 3.0 [10,11]. There is, however, a lack of consensus regarding more invasive procedures such as ESD.

In this study, we examined the rates of postoperative bleeding from post-ESD gastric ulcers following ESD for gastric tumors in accordance with a protocol specifying uniform rules for cessation and recommencement of antithrombotic therapy, in a retrospective study. We also investigated the risk factors for such bleeding in a case–control study to provide effective prophylaxis.

## Methods

### Patients

The subjects were 833 patients who underwent ESD for gastric tumors (616 with early gastric cancers, 217 with gastric adenomas) at the Osaka Medical College Hospital between June 2002 and October 2012. We use the overall term antithrombotic therapy to include therapy with antiplatelet agents (LDA, thienopyridines) and anticoagulants (warfarin potassium). Antiplatelet agents were administered as monotherapy (one or two agents) or in combination with an anticoagulant. The study included patients with a history of cerebral infarction following surgery for valvular disease. Accordingly, all patients on anticoagulants were also on antiplatelet agents, and none were on anticoagulant monotherapy. We compared hemorrhage rates between the antithrombotic and non-antithrombotic groups after ESD. We also examined risk factors for such bleeding in the antithrombotic group as a case–control study.

### ESD

We used a VIO 300 D (ERBE Elektromedizin, Tübingen, Germany) high-frequency electrosurgical generator. Approximately 5 mm outside the lesion margin, we placed markings with a needle knife (KD-1 L; Olympus Medical Systems Co. Ltd, Tokyo, Japan) [12] using a coagulation wave (Soft Coag, Effect 7, 60 W). Next we injected 0.05% adrenaline in physiological saline into the area to be excised, between the muscularis propria and the mucosa to give adequate mucosal elevation. The precut was performed using mainly the needle knife and a cutting wave (Endo Cut I, Effect 2, Duration 3, Interval 3), and the circumferential cut using mainly an IT Knife2 electrosurgical knife (KD-611 L; Olympus) [13] and a cutting wave (Endo Cut Q, Effect 2, Duration 4, Interval 3). Submucosal dissection was similarly performed using the IT Knife2, essentially using a coagulation wave (Swift Coag, Effect 2, 60–70 W) in conjunction with a cutting wave (Endo Cut Q, Effect 2, Duration 4, Interval 3) for difficult-to-dissect areas with marked fibrosis. For intraoperative bleeding, if oozing occurred, hemostasis was first attempted using a coagulation wave (Swift Coag, Effect 3, 60–80 W), still using the IT Knife2. If this was ineffective, hemostasis was achieved by pinpoint grasping of the bleeding source with hemostatic forceps (FD-410LR; Olympus) and a soft coagulation wave (Soft Coag, Effect 6, 80 W). Similarly, for arterial spurting, the hemostatic forceps were used, and if the heat was not adequately transferred to the bleeding vessel with soft coagulation, hemostatic forceps with a larger contact area (Radial Jow 3 HOT; Boston Scientific, Marlborough, USA) were substituted and hemostasis was achieved with a high-output coagulation wave (Forced Coag, Effect 2, 40 W) for 1–2 s.

Following resection of the lesion, to prevent postoperative hemorrhage all blood vessels visible in the ulcer base were treated with a coagulation wave (Soft Coag, Effect 6, 80 W) using hemostatic forceps.

### Treatment of post-ESD ulcer

**Figure 1:**
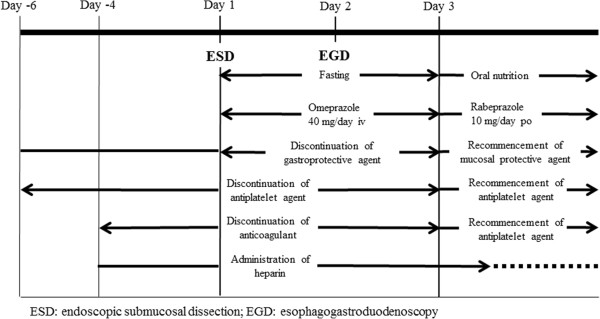
**Endoscopic submucosal dissection protocol.** For all patients, the day of the ESD procedure was set as Day 1. Patients were fasted until Day 2 and allowed to eat from Day 3. After resumption of oral feeding on Day 3, patients were commenced on an oral PPI. Patients regularly taking mucosal protective agents prior to undergoing ESD were asked to discontinue them on Days 1 and 2 and recommence them on Day 3. The protocol for antiplatelet agents was to discontinue them from Day −6 to Day 2, and that for anticoagulants was to discontinue them from Day −4 to Day 2. Heparin was substituted for anticoagulants while the latter were discontinued, maintaining the APTT at roughly twice the preheparinization level. Antiplatelet agents were recommenced as soon as possible on postoperative Day 3, following confirmation of hemostasis by EGD on Day 2. Anticoagulants were similarly recommenced on postoperative Day 3, and heparin was discontinued once the PT-INR had returned to a therapeutic level.

For all patients, perioperative management was conducted in accordance with the ESD clinical protocol of this hospital. Setting the day of the ESD procedure as Day 1, whether they were on antithrombotic therapy or not, patients were fasted until Day 2, and allowed to eat from Day 3. Intravenous PPI therapy (omeprazole 40 mg/day) was commenced immediately postoperatively. Patients underwent EGD on Day 2 to confirm hemostasis, and if necessary any blood vessels visible in the ulcer base were cauterized using a soft coagulation wave (Soft Coag, Effect 6, 80 W). After resumption of oral feeding on Day 3, patients were commenced on an oral PPI (rabeprazole 10 mg/day). Patients regularly taking mucosal protective agents prior to undergoing ESD, e.g., for chronic gastritis, were asked to discontinue them on Days 1 and 2 and recommence them on Day 3 (Figure 1).

### Guidelines for cessation and recommencement of antithrombotic therapy

After confirming with the prescribing physician whether antithrombotic therapy could be discontinued, ESD was performed only on patients able to discontinue antithrombotic therapy. The protocol for antiplatelet agents was to discontinue them from Day −6 to Day 2, and for anticoagulants to discontinue them from Day −4 to Day 2. Heparin (unfractionated heparin 10000–20000 U continuous venous infusion) was substituted for anticoagulants while the latter were discontinued, with measurement of the activated partial thromboplastin time (APTT) as appropriate and maintenance of the APTT at roughly twice the preheparinization level. In view of the risk of thromboembolic disease, antiplatelet agents were recommenced as soon as possible on postoperative Day 3, following confirmation of hemostasis by EGD on Day 2. Anticoagulants were similarly recommenced on postoperative Day 3, and heparin was discontinued once the PT-INR had returned to a therapeutic level (Figure 1).

### Definition of postoperative hemorrhage

We performed EGD for all patients on Day 2, and no patient showed bleeding. All the patients resumed taking antithrombotic agents on Day 3. Therefore, postoperative hemorrhage was defined as hematemesis and/or melena or a sudden drop in hemoglobin (Hb) ≥ 2 mg/dL occurring after recommencing eating on Day 3, requiring an unscheduled EGD, at which bleeding was confirmed to be from the post-ESD ulcer.

### Statistical analysis

Categorical data were compared using the χ^2^ test (with Yates’ correction) or Fisher’s exact test. Differences in the means of continuous data were compared using Student’s *t*-test or the Mann–Whitney *U*-test. To identify important risk factors for post-ESD bleeding, predictors with p < 0.2 in the univariate analysis were included in a backward stepwise multiple logistic regression model. p < 0.05 was considered significant, and all tests were two-sided. Data are expressed as mean ± SD or as median (range). All statistical analyses were performed using PASW Statistics 18 for Windows (SPSS Japan, Tokyo).

### Ethics considerations

All patients received oral and written explanation of the study prior to participation and gave written informed consent. The study was conducted in accordance with the Declaration of Helsinki (1995) after the protocol had been approved by the Ethics Review Committee of Osaka Medical College.

## Results

**Figure 2:**
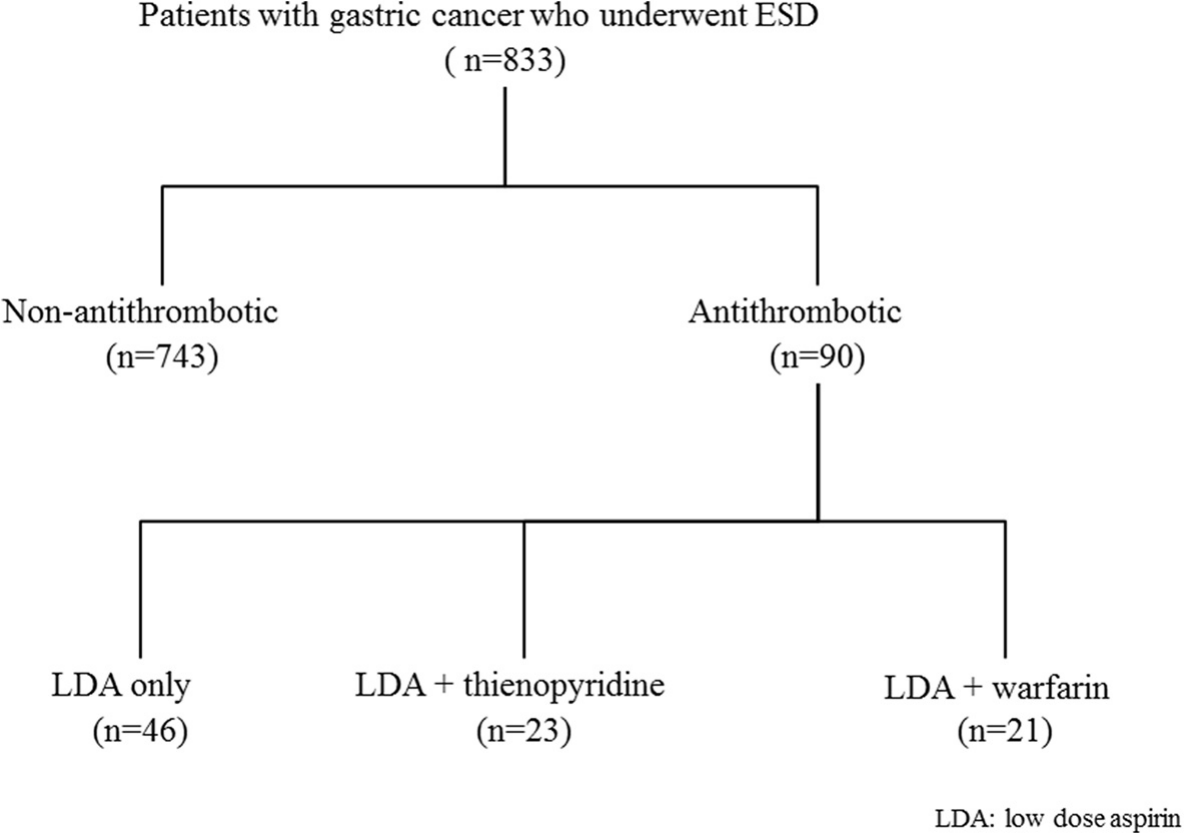
**Study outline.** There were 743 patients in the non-antithrombotic group and 90 in the antithrombotic group. The underlying disease in the antithrombotic group was a cardiac condition in 77.8% (70/90), and cerebrovascular disease in 22.2% (20/90). There were 46 patients on LDA monotherapy, 23 on LDA + thienopyridine and 21 on LDA + warfarin.

There were 743 patients in the non-antithrombotic group and 90 in the antithrombotic group. The underlying disease in the antithrombotic group was a cardiac condition in 77.8% (70/90) and cerebrovascular disease in 22.2% (20/90). There were 46 patients on LDA (Bayaspirin®) monotherapy, 23 on LDA + thienopyridine (Panaldine®, clopidogrel), and 21 on LDA + warfarin (Figure 2).

No significant differences were seen between the non-antithrombotic and antithrombotic groups in any background factors: age, gender, *Helicobacter pylori* (*H. pylori*) infection rate, tumor size, tumor site, tumor morphology, prevalence of concurrent disease (diabetes, renal failure or cirrhosis), or use of mucosal protective agents. However, the postoperative bleeding rate in the antithrombotic group was 23.3% (21/90) at a median 5.5 days (range 3–15 days), significantly higher than that of 2.0% (15/743) in the non-antithrombotic group at a median 3.5 days (range 3–10 days) (p < 0.001). There was only one reported thromboembolytic episode (1.1%), a case of cerebral infarction (Table 1).

**Table 1:** Characteristics of non-antithrombotic and antithrombotic groups

		**Non-antithrombotic (n = 743)**	**Antithrombotic (n = 90)**	**p value**
Age(yr)		65.3 ± 12.3	64.8 ± 13.7	0.719
Gender(M?F)(%M)		423/320(56.9)	54/36(60.0)	0.578
*H. pylori* infection (+/−)(%positive)		587/156(79.0)	73/17(81.1)	0.642
Tumor sixe(mm)		15.5 ± 5.2	15.7 ± 5.5	0.731
Tumor type	IIa	430	51	0.976
	IIa + IIc	112	14	
	IIc	201	25	
Duration of ESD(min)		46.6 ± 17.6	44.0 ± 16.1	0.182
Location of tumor	Antrum	325	41	0.944
	Angulus	185	22	
	Corpus	233	27	
Concrurrent disease(diabetes, renal failure, cirrhosis)(+/−)(%positive)		240/503(32.3)	28/62(31.1)	0.819
Gastroprotective agent(+/−)(%positive)		315/428(42.4)	39/51(43.3)	0.865
post-ESD bleeding(+/−)(%positive)		15/728(2.0)	21/69(23.3)	<0.001^※^

*ESD* endoscopic submucosal dissection.

^※^p < 0.05 vs non-bleeding. Ratios were analysed using the χ^2^ test.

Comparison of the 21 patients who experienced postoperative hemorrhage and the 69 who did not out of the 90 patients on antithrombotic therapy revealed no significant differences in any background factors: age, gender, tumor size, tumor site, *H. pylori* infection rate, prevalence of concurrent disease (diabetes, renal failure or cirrhosis), or number of antiplatelet agents (LDA only or LDA + thienopyridine) (p > 0.05). However, significant differences were seen according to ESD duration, LDA + warfarin combination therapy, and PPI + mucosal protective agent combination therapy (17 patients were on rebamipide 300 mg/day, 8 on teprenone 150 mg/day, 5 on ecabet sodium hydrate 2.0 g/day, and 4 on irsogladine maleate 4 mg/day) (Table 2). Multivariate analysis of these factors yielded odds ratios of 1.04 for ESD duration (95% CI 1.01–1.08, p = 0.025), 14.83 for LDA + warfarin combination therapy (95% CI 3.91–56.26, p < 0.001), and 0.27 for PPI + mucosal protective agent combination therapy (95% CI 0.07–1.02, p = 0.054) (Table 3).

**Table 2:** Background characteristics of patients by bleeding status on antithrombotic group

		**Bleeding (n = 21)**	**Non-bleeding (n = 69)**	**p value**
Age(yr)		65.4 ± 13.4	64.7 ± 13.8	0.838
Gender (M/F)(%M)		12/9(57.1)	42/27(60.9)	0.760
*H. pylori* infection(+/−)(%positive)		17/4(81.0)	56/13(81.2)	0.983
Tumor size(mm)		15.8 ± 6.1	15.7 ± 5.4	0.942
Tumor type	IIa	11	40	0.411
	IIa + IIc	2	12	
	IIc	8	17	
Ulcerative scars in the tumor(+/−)(%positive)		5/16(23.8)	16/53(23.2)	0.953
Histology (Adenoma/Well-differentiated adenocarcinoma)		3/18(14.3)	9/60(13.0)	0.883
Duaration of ESD(min)		50.3 ± 18.8	42.1 ± 14.9	0.014^※^
Location of tumor	Antrum	9	32	0.528
	Angulus	7	15	
	Corpus	5	22	
Concrurrent disease(diabetes, renal failure, cirrhosis)(+/−)(%positive)		3/18(14.3)	25/44(36.2)	0.057
Gastroprotective agent(+/−)(%positive)		5/16(23.8)	34/35(49.3)	0.039^※^
LDA + warfarin(+/−)(%positive)		12/9(57.1)	9/60(13.0)	<0.001^※^
LDA + thienopyridine(+/−)(%positive)		3/18(14.3)	20/49(29.0)	0.176

*ESD* endoscopic submucosal dissection, *LDA* low-dose aspirin.

^※^p < 0.05 vs non-bleeding. Ratios were analysed using the χ^2^ test.

**Table 3:** Significant predictors of post-ESD bleeding identified by using multiple logistic regression

**Parameter**	**Odds ratio**	**95% CI**	**p value**
Duration of ESD	1.04	1.01-1.08	0.025^※^
LDA + warfarin	14.83	3.91-56.26	<0.001^※^
PPI + gastroprotective agent	0.27	0.07-1.02	0.054

^※^p < 0.05.

*ESD* endoscopic submucosal dissection, *LDA* low dose aspirin, *PPI* proton-pump inhibitor.

## Discussion

In this study we found that, among patients undergoing gastric ESD, the risk of postoperative bleeding was higher in those on antithrombotic therapy than in those not on antithrombotic therapy. Although there was no significant difference between postoperative bleeding rates in the LDA monotherapy and LDA + thienopyridine combination therapy groups, LDA + warfarin combination therapy was an extremely strong risk factor for post-ESD bleeding. In the study, we investigated for the first time the risk of hemorrhage following gastric ESD in patients on antithrombotic therapy on the basis of a protocol setting out the timing of their discontinuation and recommencement of antithrombotic therapy, as well as the risk associated with different antithrombotic agents.

The American Society for Gastrointestinal Endoscopy guidelines for the management of antithrombotic agents for endoscopic procedures published in 2009 recommend that LDA therapy be continued for gastrointestinal endoscopies, even for procedures with a high risk of hemorrhage [14]. On the other hand, the corresponding European Society of Gastrointestinal Endoscopy guidelines published in 2011 state that, in principle, LDA should be continued for most endoscopies but recommend cessation of LDA for 5 days for ESD and other procedures with a high risk of hemorrhagic complications, provided the risk of thromboembolic events is low [15].

In this study, after confirming with the prescribing physician that antithrombotic agents could be discontinued, we performed ESD on patients at low risk of thromboembolic events following a set period of discontinuation of antithrombotic agents. There was only one reported thromboembolytic episode (1.1%) attributable to cessation of antithrombotic therapy. When a patient on LDA therapy discontinues aspirin for about 4 weeks, the reported odds ratio for stroke or transient ischemic attack is 3.29 (95% CI 1.07–9.80, p < 0.005) [16]. In this study, we discontinued LDA either for a shorter period or not at all, so as to keep the incidence of cerebral infarction to a relatively low level. However, the postoperative bleeding rate in the antithrombotic group was 23.3%, significantly higher than the 2.0% observed in the non-antithrombotic group.

There is a lack of clear evidence of the extent to which hemorrhagic complications are increased in patients on antiplatelet + anticoagulant combination therapy undergoing gastrointestinal endoscopic procedures with a high risk of bleeding. In a retrospective study of 5593 patients undergoing colorectal polypectomy, postoperative hemorrhage was significantly more common in patients on warfarin therapy [17]. From this, it goes without saying that warfarin should be discontinued for colorectal polypectomy, and the recommendation for ESD, with its high incidence of hemorrhagic complications, is to discontinue warfarin and replace it with heparin [18]. We also replace warfarin with heparin, monitoring APTT as we perform the procedure. Patients on warfarin therapy undergoing ESD in this study included some with a history of cerebral infarction following surgery for valvular disease, so all patients on warfarin were also on LDA. Because there were no patients on warfarin monotherapy in this study, it is uncertain whether the risk of postoperative bleeding was increased by LDA + warfarin combination therapy or by warfarin alone. However, the postoperative bleeding rate was significantly higher in patients on LDA + warfarin combination therapy, with an odds ratio of 14.83 (p < 0.001), confirming combination therapy to be an extremely strong risk factor. The odds ratio for postoperative bleeding in patients taking mucosal protective agents in addition to a PPI was 0.27 (p = 0.054), suggesting that the addition of a mucosal protective agent may be effective in preventing postoperative hemorrhage, although the difference was not significant. To date, only four studies worldwide have examined the relationship between antithrombotic therapy and bleeding following gastric ESD.

In 2009, Ono et al. [19] published a retrospective study of gastric ESD in patients on antithrombotic therapy. They reported a postoperative bleeding rate of 10.7% (6/56) in patients on antithrombotic therapy and 5.2% (20/388) in patients not on antithrombotic therapy, with no significant difference between the groups. All patients on antithrombotic therapy were also taking an antiplatelet agent, with 5 on combination therapy with an anticoagulant. Of these, only 3 were heparinized. All patients discontinued antithrombotic therapy for 1 week before and after ESD. The reason for the lack of a significant difference in the postoperative bleeding rate between patients on antithrombotic therapy and those not on antithrombotic therapy may be because the drug withdrawal period was longer than for the present study. Considering the risk of thromboembolic events, however, antithrombotic therapy should be recommenced as soon as possible, once hemostasis has been confirmed endoscopically. The standards for cessation and recommencement of antithrombotic therapy, as well as heparinization, are vague and ambiguous.

In 2010, Mannen et al. [20] published a retrospective study of risk factors for complications following ESD in 436 patients with gastric tumors. They reported a postoperative bleeding rate of 3% (1/33) in patients on antithrombotic therapy and 9.4% (38/403) in patients not on antithrombotic therapy, with no significant difference between the groups.

In a similar retrospective study published in 2011, Okada et al. [21] reported risk factors for post-ESD bleeding in 582 patients with gastric tumors. They reported a postoperative bleeding rate of 14.2% (4/28) in patients on antithrombotic therapy and 12.6% (70/554) in patients not on antithrombotic therapy, with no evidence for a causal relationship between postoperative bleeding and antithrombotic therapy (p = 0.7732).

However, these studies do not report in detail whether patients were on antiplatelet agents only or combination therapy with an anticoagulant, the timing of cessation and recommencement of antithrombotic therapy, or whether heparin was substituted.

A study that found that antithrombotic therapy increases the risk of postoperative bleeding was the 2010 retrospective study by Tsuji et al. [22] of gastric ESD in patients on antithrombotic therapy, as well as corticosteroids and nonsteroidal anti-inflammatory drugs (NSAIDs). They reported a postoperative bleeding rate of 34.8% (8/15) in patients on antithrombotic therapy and 16% (60/315) in patients not on antithrombotic therapy, significantly higher in the former with an odds ratio of 2.76 (95% CI 1.09-6.98). As in the present study, they followed a protocol with set timing of cessation and recommencement of antithrombotic therapy, and heparinization for patients on anticoagulant therapy, and they reported an increased risk of post-ESD bleeding in patients on antithrombotic therapy. However, corticosteroids and NSAIDs are included among the antithrombotic agents, and antithrombotic agents are not examined separately, so simple comparisons cannot be made.

On the other hand, suppressors of acid secretion, histamine type-2 receptor antagonists and PPIs, were used to treat post-ESD ulcers in the above four studies, but gastroprotective agents were not used. In this study, although multivariate analysis did not show a significant difference (odds ratio: 0.27, p = 0.054) in combination therapy with a mucosal protective agent and a PPI, univariate analysis showed significant differences (p = 0.039) between the two groups. For the first time we added these results suggest that the addition of a mucosal protective agent may be effective in preventing post-ESD hemorrhage in patients on antithrombotic therapy.

In the treatment of hemorrhagic peptic ulcers, suppressors of acid secretion such as PPIs promote ulcer healing as well as reducing the risk of hemorrhage [23,24]. On the other hand, in the treatment of post-ESD ulcers, PPI + mucosal protective agent combination therapy is reported to yield better healing rates and similar postoperative bleeding rates as PPI monotherapy [25,26]. These studies report no significant difference between postoperative bleeding rates in the PPI group and PPI + mucosal protective agent group, but the studies were conducted with patients not on antithrombotic therapy. In this study, we included patients on antithrombotic therapy, and the fact that our results show a reduction in postoperative bleeding can be attributed to a superior promotion of ulcer healing with PPI + mucosal protective agent combination therapy, as demonstrated in the earlier studies.

Asian people are generally considered to have lower gastric acid levels than Westerners [27-29], and furthermore differentiated type gastric cancers, one of the indications for ESD, show a markedly atrophic background mucosa associated with *H. pylori* infection. From this we can assume impairment of gastric mucin and other protective factors, as well as reduced acid secretory function. We can also infer that the pharmacological properties of mucosal protective agents exert favorable effects on the post-ESD healing process, including prevention of postoperative hemorrhage.

The tendency towards higher postoperative bleeding rates with longer ESD durations can be explained in terms of a longer time taken to achieve hemostasis while resecting the lesion. When multiple vessels require cautery, it follows that a number of vessels are present in the ulcer floor following ESD, and we can assume that this influences the postoperative bleeding rate.

The limitations of this study are that it was a retrospective study, that we did not perform ESD while continuing antithrombotic therapy, the absence of a warfarin monotherapy group, and the possibility of a bias in the administration of mucosal protective agents. We are a tertiary referral center, resulting in a relatively high proportion of patients with background factors such as cerebral infarction following valvular surgery. All patients on warfarin therapy were also taking LDA, so that we had no warfarin monotherapy group for comparison. We were unable to identify any earlier studies that examined this area in detail based on a consistent protocol, and we believe the next step should be to conduct a prospective study on the basis of our results. PPI monotherapy is the standard treatment for post-ESD ulcers, but the risk of postoperative hemorrhage cannot be avoided through a PPI alone in patients on antithrombotic therapy. Our results indicate that the addition of a mucosal protective agent to PPI therapy may reduce the risk of postoperative hemorrhage, potentially an extremely useful finding. We intend to conduct a large-scale prospective trial to confirm this.

## Conclusions

The risk of postoperative hemorrhage following gastric ESD was higher in patients with antithrombotic therapy than in those without that therapy. Among these patients, LDA + warfarin combination therapy and longer ESD duration were significant risk factors for postoperative bleeding. On the contrary, a mucosal protective agent to PPI therapy, lowering the odds ratio for postoperative bleeding, which suggests that the addition of a mucosal protective agent might be effective in preventing post-ESD hemorrhage in patients on antithrombotic therapy. A further prospective study with a large sample will be needed to confirm these.

## Abbreviations

ESD: Endoscopic submucosal dissection; LDA: Low-dose aspirin; PPI: Proton-pump inhibitor; PT-INR: Prothrombin time-international normalized ratio; APTT: Activated partial thromboplastin time; Hb: Hemoglobin; H. pylori: *Helicobacter pylori.*

## Competing interests

The authors have no conflicts of interest to declare.

## Authors’ contributions

Guarantor of the article: TT. Specific author contributions: Principal investigator, subject evaluation, data collection and manuscript preparation: TT; manuscript preparation and statistical analysis: KH; subject evaluation and data collection: KO, SH, SE, ST and EU. All authors read and approved the final manuscript.

## Pre-publication history

The pre-publication history for this paper can be accessed here:

http://www.biomedcentral.com/1471-230X/13/136/prepub
